# An On-Chip RBC Deformability Checker Significantly Improves Velocity-Deformation Correlation

**DOI:** 10.3390/mi7100176

**Published:** 2016-10-01

**Authors:** Chia-Hung Dylan Tsai, Junichi Tanaka, Makoto Kaneko, Mitsuhiro Horade, Hiroaki Ito, Tatsunori Taniguchi, Tomohito Ohtani, Yasushi Sakata

**Affiliations:** 1 Department of Mechanical Engineering, Osaka University, Suita 565-0871, Japan; tanaka@hh.mech.eng.osaka-u.ac.jp (J.T.); mk@mech.eng.osaka-u.ac.jp (M.K.); horade@mech.eng.osaka-u.ac.jp (M.H.); ito@hh.mech.eng.osaka-u.ac.jp (H.I.); 2 Department of Cardiovascular Medicine, Osaka University the Graduate School of Medicine, Suita 565-0871, Japan; taniguch@cardiology.med.osaka-u.ac.jp (T.T.); ohtani@cardiology.med.osaka-u.ac.jp (T.O.); sakata@medone.med.osaka-u.ac.jp (Y.S.)

**Keywords:** red blood cells (RBC), deformability, velocity–deformation correlation, microfluidic constriction

## Abstract

An on-chip deformability checker is proposed to improve the velocity–deformation correlation for red blood cell (RBC) evaluation. RBC deformability has been found related to human diseases, and can be evaluated based on RBC velocity through a microfluidic constriction as in conventional approaches. The correlation between transit velocity and amount of deformation provides statistical information of RBC deformability. However, such correlations are usually only moderate, or even weak, in practical evaluations due to limited range of RBC deformation. To solve this issue, we implemented three constrictions of different width in the proposed checker, so that three different deformation regions can be applied to RBCs. By considering cell responses from the three regions as a whole, we practically extend the range of cell deformation in the evaluation, and could resolve the issue about the limited range of RBC deformation. RBCs from five volunteer subjects were tested using the proposed checker. The results show that the correlation between cell deformation and transit velocity is significantly improved by the proposed deformability checker. The absolute values of the correlation coefficients are increased from an average of 0.54 to 0.92. The effects of cell size, shape and orientation to the evaluation are discussed according to the experimental results. The proposed checker is expected to be useful for RBC evaluation in medical practices.

## 1. Introduction

The relations between the deformability of human cells, especially red blood cells (RBCs), and diseases have been investigated and reported in the last few decades [1,2,3]. For example, Nash et al. found that RBCs from the patients with malaria lose their deformability [4]. Reid et al. showed a significant difference of RBC deformability between patients with peripheral vascular disease and normal subjects [5]. Among all different cell evaluations, the evaluation with a constriction channel in a microfluidic device has become one of the most popular approaches because of its simplicity and high-throughput. The idea of such microfluidic approaches is to let cells pass through a constriction channel whose cross-section area is smaller than the size of the cells. Therefore, the cells have to deform in order to pass through, and different cell deformability would result in different passing velocities during the transit [6]. For example, a relatively faster transit velocity would be obtained if the passing cell is relatively softer, and vice versa [7,8]. However, correlations between transit velocity and cell deformation are usually not very solid (|R|<0.7), and are sometimes even weak (|R|<0.5), due to the limited range of cell deformation. The proposed RBC deformability checker aims to resolve the problem of the low correlation and to advance RBC evaluation in medical practices.

**Figure 1:**
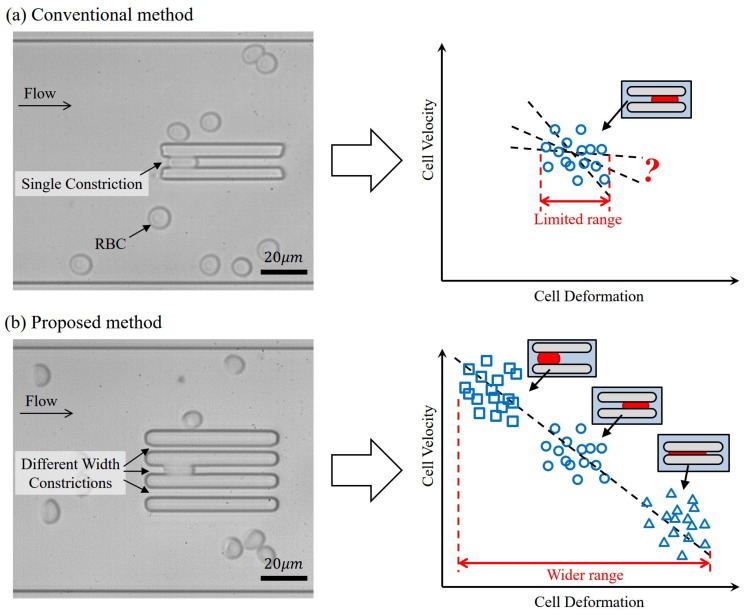
A comparison between a conventional method and the proposed method. (**a**) In conventional methods, the range of cell deformation is limited due to single constriction channel. Reprinted with permission from [10]; (**b**) The proposed method imparts a wider range of deformation by the constrictions of different widths, which are around 3.5, 4.0, and 4.5 μm from the top to the bottom.

Figure 1 demonstrates the comparison between the conventional and proposed constriction approaches. In the conventional approach, as an example shown in Figure 1a, all evaluated cells are passed through the same constriction on a microfluidic chip, and the amount of cell deformation and its motion as transit time or velocity are measured for the evaluation of cell deformability [6,7,8,9]. The relation between the deformation and cell velocity is illustrated on the right of Figure 1a. The *x*- and *y*-axes are the amount of cell deformation and cell transit velocity through the constriction, respectively. Negative correlation between the deformation and the transit velocity is expected because a stronger resistance force against cell moving forward is generated when a greater deformation is applied to a cell [7]. The variation of cell deformation is from the variation of cell size, that is, a large cell would deform more through the same constriction. If the correlation is weak, as the illustrated on the right of Figure 1a, the evaluation results cannot provide a clear tendency of cell deformability. Thus, it is difficult to assuredly tell the difference of cell deformability from one sample to another unless taking a very large sample size for statistical significance.

Figure 1b shows the proposed deformability checker and expected results. Three constrictions of different widths, around 3.5, 4.0, and 4.5 μm from the top to the bottom in this case, are placed in parallel on a microfluidic chip. The three constrictions introduce three different deformation regions as illustrated on the right of Figure 1b. A wider range of deformation can be achieved by considering three regions as a whole. The extended range of cell deformation gives a more comprehensive information of cell deformability because a clearer tendency of cell behavior can be seen, and significant difference of cell velocities from small deformation to big deformation can be obtained.

Experiments on human RBCs were performed for validating the proposed deformability checker. RBCs from five volunteer subjects were tested. It was found that the proposed method consistently improves the correlations between transit velocity and deformation of the RBCs compared with single constrictions. The absolute values of the correlation coefficients are increased from an average of 0.54 for single constriction to an average of 0.92 for the proposed three constrictions. The cell size, flow-in shape and orientation are discussed based on the experimental results.

The rest of this paper is structured as follows: after a brief review on related literature in Section 2, the proposed idea and design will be explained in Section 3, and experimental setup, procedures and results are presented in Section 4 before the discussions of cell size, shape and orientation in Section 5. Finally, the concluding remarks are summarized in Section 6.

## 2. Related Works

Different approaches for evaluating cell deformability have been developed [11,12,13,14]. For example, Radmacher et al. measure the viscoelastic properties of human platelets with an atomic force microscope (AFM) [15]. Brandao et al. use optical tweezers for realizing the mechanical characterization of human RBCs [16]. Among different approaches, microfluidic devices provide a convenient way to evaluate single cell deformability at high throughput and in a clean environment [7,17,18,19,20,21,22]. For example, Otto et al. developed a real-time deformability cytometry for on-the-fly cell phenotyping [23]. Gossett et al. achieved the rate of 2000 cells per second by hydrodynamic stretching [24]. Parallel constrictions, which are similar to the structure of the proposed design, have been previously developed for different purposes [25,26]. For example, Gifford et al. used parallel microchannels with different shapes for measuring the mean corpuscular volume of individual RBCs [27]. Young et al. investigated endothelial cell adhesion by a parallel microfluidic network for different coating conditions [28].

To the best of the authors’ knowledge, the proposed deformability checker is the first work aiming to improve the velocity–deformation correlation by widening the range of cell deformation. The idea of the checker is simple and straightforward. According to the experimental results, the method significantly improves the correlations between the transit velocity and cell deformation. It is believed that the proposed deformability checker could benefit the RBC evaluation in medical practices.

## 3. The Proposed Method

**Figure 2:**
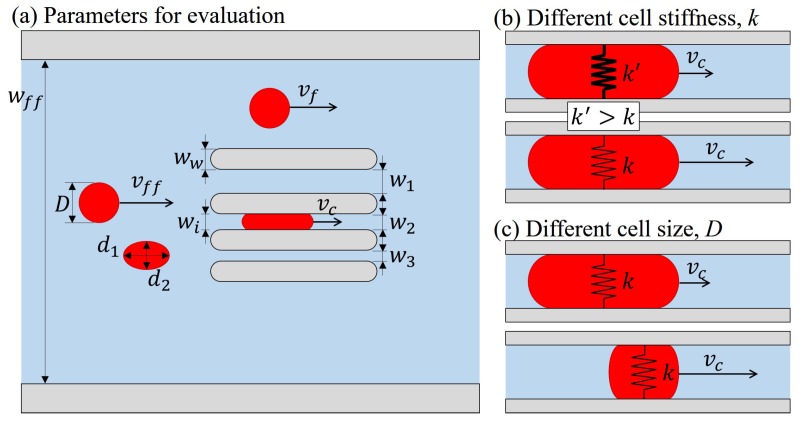
The parameters for deformability evaluation. (**a**) The cell size and velocity before and after entering the constrictions are measured; (**b**) transit velocities of two cells with different stiffness; and (**c**) transit velocities of two cells with different cell sizes.

Figure 2a shows the proposed method that includes three parallel constrictions of different widths. Cells are suspended and moved with the flow inside the microfluidic channel, and they randomly enter any of the constrictions depending on their position in the flow. Two by-pass channels, the wide paths on both sides of the constrictions, are for maintaining a stable pressure drop across the constrictions, and they are important for the consistency of the evaluation. The by-pass channels have been proved theoretically and experimentally, and have been widely used for microfluidic applications [10,29,30].

The key idea of the proposed deformability is to extend the range of cell deformation by the three constrictions. Each constriction generates a region of cell deformation because of different geometric constraints to the test cells. For example, if two RBCs of the same size pass through the wide and narrow constrictions together, the one through the narrow constriction would deform more than the one through the wide constriction. In order to fairly compare cell deformation from cells with different undeformed sizes, the cell deformation, ϵ, is normalized as the ratio between the amount of deformation and its original size. The normalization is also a well-known parameter as mechanical strain in general material tests. The normalized cell deformation can be expressed as

(1) $$\epsilon =\frac{\Delta D}{D}\;\mathrm{where}\;\Delta D=D-w_{i}$$

where Δ*D*, *D*, and $w_{i}$ are the amount of deformation, the diameter of undeformed RBCs and the width of *i*th constriction, respectively. If an RBC is not perfectly circular from the captured image, the diameter, *D*, is calculated from the length of two principal axes of the RBC as $D=\sqrt{d_{1}d_{2}},$ where $d_{1}$ and $d_{2}$ are the first and second principal axis as illustrated in Figure 2a. A greater ϵ indicates a greater deformation. The three constrictions result in a wider range of cell deformation, and it can be quantitatively justified with the defined cell deformation ϵ—for example, if the constriction widths are 3.5, 4.0, and 4.5 μm, and cell diameter ranges from 6 to 8 μm. The cell can be deformed from 25% to 56%, and the variation of deformation is 31%. On the other hand, if the same group of cells are tested by a single constriction whose width is 4.0 μm, the cells can only be deformed from 33% to 50%, where the variation of deformation is only 17%, about the half of the range of the three constrictions.

On the other hand, the transit velocity $\hat{v}$ of an RBC through a constriction is normalized by the flow velocity, and is defined as

(2) $$\hat{v}=\frac{v_{c}}{v_{f}}$$

where $v_{c}$ and $v_{f}$ are the velocity of a cell through a constriction and fluid velocity on the same cross section, respectively. The theoretical range of the transit velocity is from 0 to 1 because of the normalization. Any value of the transit velocity, $\hat{v}$; below one is assumed to be caused by the resistance coming from the interaction between constrictions and deformed RBCs during the transit. Since local fluid velocity cannot be directly observed, the fluid velocity is determined using undeformed cells as flow markers, and cell velocity outside the constrictions are considered the same as the flow velocity. The fluid velocity on the same cross-section as the constrictions is represented by $v_{f}$, which is calculated from cell velocity before entering the constrictions as $v_{ff}$ under the assumption of incompressible flow. The assumption gives the constant volumetric flow rate at any cross-section of the fluid flow, and $v_{f}$ can then be obtained as

(3) $$v_{f}=v_{ff}\frac{w_{ff}}{\left(w_{ff}-4w_{w}\right)}$$

where $v_{ff}$, $w_{ff}$ and $w_{w}$ are the cell velocity before entering the constrictions, the width of the overall microchannel, and the width of constriction walls. The parameters are illustrated in Figure 2a.

Figure 2b,c show examples of how transit velocity being affected by cell stiffness and cell size, respectively. Figure 2b shows an example of two passing cells with different stiffness, *k*. The cell stiffness is associated with the flow resistance when the cell is passing through a constriction. A stiffer cell would experience a stronger resistance, which results in a slower transit velocity. Figure 2c is another example where two cells have the same stiffness, *k*, but different sizes, *D*. Cell size determines the amount of deformation while passing through a constriction. Because the contact force between a deformed RBC and the constriction cannot be directly measured, cell transit velocity is utilized as an index of contact force. Simulations on the internal stress for an RBC through a slit can be found in the literature [31]. Therefore, the velocity of a large cell is expected to be slower than a small cell due to different amounts of deformation. This example also explains the reason why a negative correlation between cell velocity and deformation is expected. Theoretical modeling and experimental supports of the examples in Figure 2b,c can be found in conventional approaches [6,7].

## 4. Experiments

### 4.1. Experimental System

**Figure 3:**
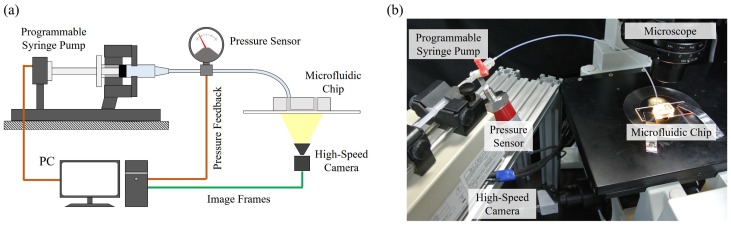
The experimental system. (**a**) A diagram of the experimental setup; and (**b**) a photo of the system.

Figure 3a,b show an overview of the experimental system and a photo of the actual experimental setup, respectively. The experimental system includes three main parts, which are pressure control system, microfluidic chip and high-speed vision system. The pressure control system provides a constant pressure source for a stable fluid flow in the microfluidic chip, and is constructed by a programmable syringe pump with a servo actuator (RSF supermini, Harmonic Drive LLC, Tokyo, Japan) and a pressure sensor (FP101A, Copal Electronics Co., Tokyo, Japan). The pressure is controlled by a feedback controller using a Proportional-Integral-Derivative (PID) algorithm. The microfluidic chip is fabricated with the proposed three constrictions of different width inside. The inlet and outlet of the microfluidic device are connected to the pressure control unit and open to the atmosphere, respectively. The details of the chip fabrication and the calibration of constriction width will be explained in the next section. The high-speed vision system is for observing the cell motion through the constrictions, and it includes a high-speed camera (IDP, Photron Co., Tokyo, Japan) and a microscope (IX71, Olympus Co., Tokyo, Japan). The high-speed camera is set at the recording rate of 3000 frames per second (fps) in experiments. The objective lens of 40× is used, and one pixel in the image represents 240 nm, which is calibrated by a micrometer.

### 4.2. Microchip Fabrication and Width Calibration

The fabrication of the microfluidic chip is done by the standard molding process and is briefly explained as follows: The constriction widths of 3.5, 4.0, and 4.5 μm, and the length of 50 μm are chosen based on our experience from previous studies [7]. Since RBC diameter usually ranges from 5 to 8 μm, the widths are expected to deform all the passing RBCs but not to have the cells stuck inside the channel. The constriction length is to provide enough tracking points of cell motion in a constriction for a fair velocity analysis. The proposed design is patterned on a silicon wafer through a photolithography drawing machine (PLS-1010, PMT Corp., Fukuoka, Japan). Before the photolithography process, the wafer is pre-coated with 3.5 μm-thick photoresist (SU8-3005, Nipponkayaku Co., Tokyo, Japan). The patterned wafer is developed as the mold for later polydimethylsiloxane (PDMS) replica. PDMS and curing agent are mixed at the ratio of 9:1, and are poured onto the mold in a container. The PDMS and mold are put into a 80 °C oven for cure. After 40 min, the PDMS is cured, and is peeled off from the mold. Two holes are punched on the PDMS chip for the inlet and outlet. Finally, the PDMS chip is bounded to a clean glass substrate using a plasma machine (CUTE, Femto Science Inc., Gyeonggi-Do, Korea).

**Figure 4:**
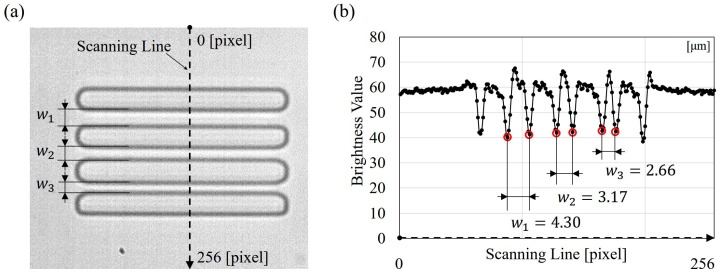
The calibration of constriction width by brightness-based method. (**a**) An image obtained through the microscope and camera with a scanning through the constrictions and by-pass channels; and (**b**) the brightness values along the scanning line in (**a**). The value ranges from 0 to 255, where 0 and 255 are black and white, respectively.

The dimensions of microfluidic devices often include errors for several hundred nanometers because the limit of the fabrication process as well as the resolution of used photoresist. The width of the constrictions is especially important in the proposed deformability checker since the width, $w_{i}$, is used for calculating cell deformation as in Equation (1). To cope with possible error in the fabrication, an image-based width measurement and a laser microscope were utilized for width measurement and cross-check. Figure 4 shows an example of the width measurement by the brightness-based method. Figure 4a,b are a snapshot of the three constrictions from the vision system and the brightness values along the scanning line in Figure 4a. The brightness value is ranged from 0 to 255 as in a standard 8-bit greyscale image, where the brightness of 0 and 255 indicates black and white, respectively. The brightness values along the scanning line in Figure 4a, from top to bottom, are retrieved for determining the width of the constrictions, and are shown in Figure 4b. Several low peaks can be found in Figure 4b, which are the local darkest points that appeared along the scanning line. The constriction widths are measured based on the distance between the peaks. The conversion from image pixel to actual distance is 0.24 μm/pixel, which is calibrated by an objective micrometer (AX001, Olympus Co.). In the example shown in Figure 4, the constriction widths are 4.30, 3.17, and 2.66 μm, which are different from the designated widths 4.5, 4.0, and 3.5 μm in the original chip design. The measurement is performed each time during the data analysis.

**Figure 5:**
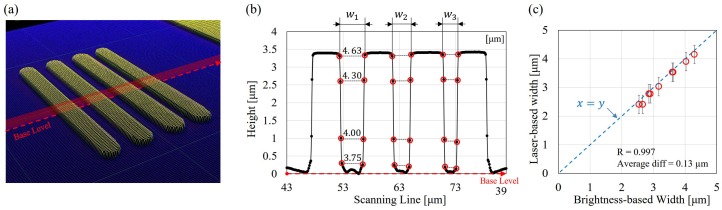
The calibration of constriction width by a laser microscopy. (**a**) A snapshot of the measurement screen of the laser microscope; (**b**) the measured channel width and height along the scanning surface; and (**c**) the comparison of width measurement between the brightness-based method and a laser microscope.

The constriction width is also measured by a laser microscope (OLS4100, Olympus Co.) for verifying the brightness-based measurement shown in Figure 4. Figure 5 shows measured results on a PDMS chip before it is bounded to a glass substrate. Figure 5a is a snapshot of the laser microscope screen during the measurement, and it shows that the layout of the channel design can be clearly identified even though the PDMS is semi-transparent. Figure 5b is an example of the measured results along the red scanning surface in Figure 5a. Each constriction width is measured as the average of multiple same level points as shown in Figure 5b. For example, constriction channel $w_{1}$ is measured as the average of 4.63 , 4.30, 4.00, and 3.75 μm from the top to the bottom. The average constriction width is 4.17 μm in Figure 5b, while the exact same chip is measured at 4.30 μm by the brightness-based method in Figure 4. The difference is 0.13 μm from the two methods in this case. Figure 5c shows the comparison between the brightness-based measurement and the measurement by the laser microscope. Three sets of parallel constrictions are measured by both methods, and average difference between the two methods is 0.17 μm, while the correlation between two measurement is up to 0.997. The red circles and error bars in Figure 5c represent the average measurement of the laser microscope and the corresponding standard deviations. A dashed line is drawn for indicating the equal measurement between the two methods. The measured points are all close to the line, which shows that the brightness-based method matches the laser microscope well, and is reasonable.

### 4.3. Experimental Procedure

RBC samples from five male subjects are tested in an experiment for validating the proposed deformability checker. All the subjects have read and agreed to the consent of the experiments prior to the test. The blood is withdrawn by a licensed physician one hour before each test. The blood is diluted by standard saline at the blood–saline ratio of 1:50 before putting it into the microfluidic chip. The dilution is necessary because a microliter of blood contains about 5 million RBCs, and single cell evaluation is not possible without such a dilution. The microfluidic chip is filled with standard saline before the diluted blood samples are injected into the chip from the inlet. After the sample injection, the inlet is connected to the pressure control system to start a constant flow driven by a pressure difference of 0.7 kPa. The motion of RBCs through constrictions are recorded by the high-speed camera for analysis. The analysis is firstly done using automatic image processing on the recorded videos [32], and then the RBC velocities and sizes are manually confirmed one by one. The confirmation is to assure the validity of the data for avoiding false detections.

### 4.4. Experimental Results

**Figure 6:**
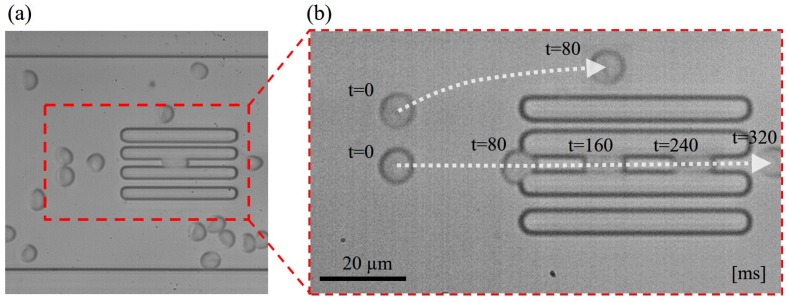
Snapshots of red blood cells (RBCs) in the microfluidic channel during the experiments. (**a**) RBCs are deformed to different amounts by different constrictions; and (**b**) an example of an RBC through a constriction in a stacked image from multiple frames.

Figure 6a shows the snapshot of RBCs and the constriction channels during the experiment, and Figure 6b is a stacked image from multiple frames showing RBCs flowing through a constriction and by-pass channel. The two RBCs in Figure 6b moved at almost the same velocity before reaching the constrictions. The RBC through the constriction took about 0.2 s for squeezing into it, while, at the same time, the by-pass RBC on the top had already flowed away. It shows that the RBC moved slower inside the constriction than outside according to the shorter distance between the RBCs at the constant time intervals in Figure 6b. The change of cell velocity is believed due to the resistance between the deformed RBC and the constriction, and it is the fundamental working principle of the evaluation method as explained in Section 3. The throughput depends on the actual flow condition and ranges from 84 to 138 cell/min from our results. The throughput is not particularly high compared with a general microfluidic approach due to two reasons. First, a relatively low pressure (0.7 kPa) is applied to drive the flow for preventing unnecessary RBC deformation from the flow shear. The other reason is that we employed a by-pass channel for stabilizing the pressure drop across the constrictions. One drawback of the by-pass channels is that most of the RBCs would pass through them instead of entering the constrictions. In order to keep stable pressure and reliable results, the by-pass channel is believed to be a necessary sacrifice for the throughput. A sample video of RBCs passing through the constrictions can be found in the Supplementary Material. The difference of RBC velocities can be clearly observed in the video S1.

**Figure 7:**
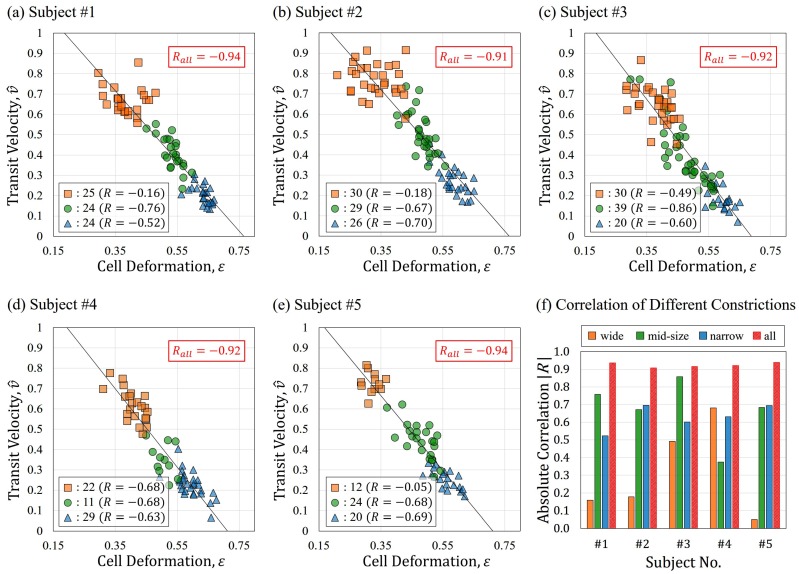
The experimental results. (**a**–**e**) The experimental results from five subjects in normalized velocity–deformation plots. Each mark represents a measured RBC through a constriction, and the rectangles, circles and triangles indicate the RBC passing through the wide, mid-size and narrow constrictions, respectively. The sample size of each constriction is shown in the lower-left corner along with its velocity–deformation correlation; (**f**) The comparison of correlation between single constrictions and proposed method which considers data from all three constrictions as a whole. The correlations of all data are consistently greater than the single constriction channel.

Figure 7 shows the analysis results of the five subjects in Figure 7a–e, and a comparison between the correlation of individual constrictions and three constrictions as a whole are presented in Figure 7f. In Figure 7a–e, the data points collected using wide, mid-size and narrow constrictions are indicated by squares, circles and triangles, respectively. The *x*- and *y*-axes are the cell deformation, ϵ, and transit velocity, $\hat{v}$, respectively. The cell deformation is calculated based on equivalent diameter, *D*, of RBCs and the constriction width, $w_{i}$, using Equation (1) where the constriction width is measured by an image-based method as described in Section 4.2. The transit velocity, $\hat{v}$, is the normalization of cell velocity with the fluid velocity, as in Equation (2), where the cell velocity, $v_{c}$, is determined only when a RBC is fully entered and deformed in the constriction.

The sample size for each constriction is shown at the lower-left in the plots of each subject along with its velocity–deformation correlation $R_{i}$ (i=1,2,3). The results by the individual constrictions are used for demonstrating a conventional single-constriction approach. All of the evaluated points of each subject are also considered as a whole, and the overall correlation is shown in the upper-right corner of each plot. The correlations including all three constrictions are significantly improved from the results obtained by each single constriction. For example, the correlations of the subject #1 are $R_{1}=-0.16$, $R_{2}=-0.76$ and $R_{3}=-0.52$ by wide, mid-size and narrow constrictions alone, while the correlation is significantly improved to $R_{\mathrm{all}}=-0.94$ by putting together all three pieces of data. The same tendency of such significant improvements of correlation is found in all five results, as shown in Figure 7f. There is no clear tendency of using any of individual constriction for obtaining consistent high correlation among the five subjects. For example, mid-size constriction provides moderate correlations (|R|>0.5) for subjects #1, #2, #3 and #5, while it failed in subject #4 with only $R_{2}=-0.38$.

According to Figure 7f, all of the absolute values of the correlation coefficients from three constrictions among the five tested subjects are always greater than 0.9. We also calculated the correlations with only the data from two constrictions, the wide and narrow ones. The correlations with the two constrictions are −0.94, −0.92, −0.93, −0.96 and −0.96 for subjects #1 to #5, respectively. The absolute values of the correlations are also consistently greater than 0.9, and it shows that two constrictions may be another workable approach. Nevertheless, the three-constriction approach still has the advantage of showing the continuous changes of cell response as deformation increases. In addition, the overlaps between the data points from constriction to constriction demonstrate the repeatability of cell response, that is, cell velocities, $\hat{v}$, are similar as long as they experience similar deformation, ϵ, regardless of different constrictions. Overall, the results indicate a strong correlation between the transit velocity and cell deformation, and cannot be seen with single constrictions. The high correlations explicitly support the proposed method successfully extracting representative characteristics of RBCs.

## 5. Discussion

Further investigation of RBCs are performed in addition to the velocity–deformation correlation. Through the recorded videos from the high-speed camera, we can extract cell properties such as RBC undeformed size, shape and orientation. These properties will be discussed as follows.

### 5.1. The Effect of RBC Size Distribution

**Figure 8:**
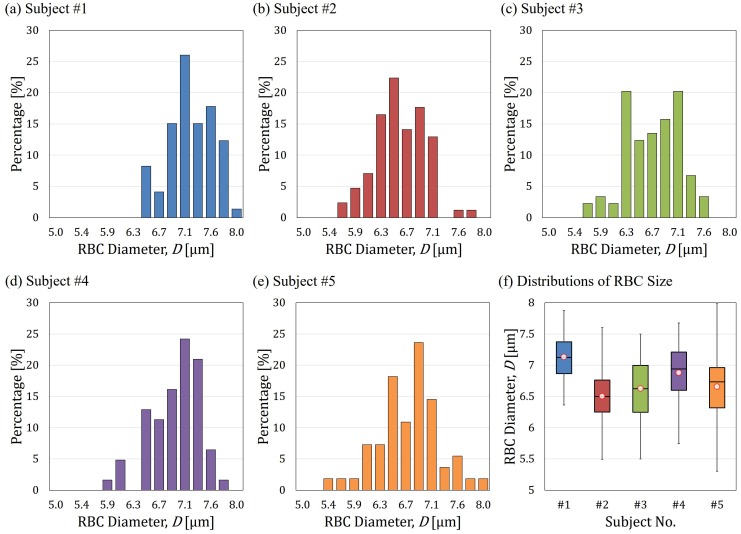
The size distribution of tested RBCs from the five subjects. (**a**–**e**) The distribution of Subject #1 to #5; and (**f**) an overview of RBC size distribution among the subjects.

Figure 8a–e show the distributions of the RBCs diameters from five subjects, and Figure 8f shows a comparison of these distributions in a box plot. All of the RBCs are within a reasonable range of human RBC as in the range of 5 to 8 μm. The size distributions can be used to interpret two unanticipated points in Figure 7. The first unanticipated point is the distribution of the RBCs through mid-size constriction in Figure 7c. Typically, the distributions of data points through the same constrictions are close to each other, but the distribution of the data by mid-size constriction in Figure 7c forms two groups, that is, one at the center and the other in the upper-left corner. A possible interpretation is that there are two peaks in the distribution of cell size for the subject #3 in Figure 8c. The uneven distribution of cell size may be a reason for forming the two groups. The second unanticipated point is that the correlation of mid-size constriction in Figure 7d is unexpectedly low. A clear absence of cell diameter around 6.3 μm is found in Figure 8d, and may be the cause of the problem.

### 5.2. The Effect of RBC Shape and Orientation

**Figure 9:**
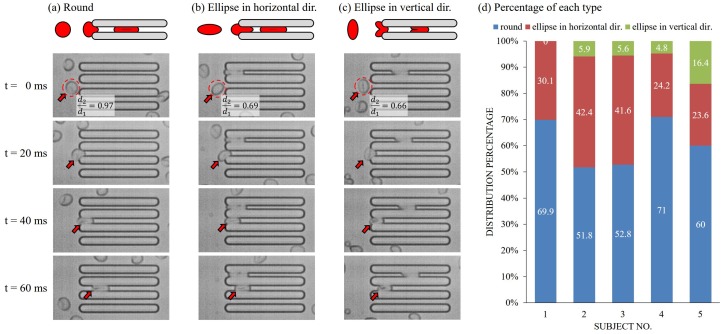
Different initial shape and orientation of RBCs are found during analysis. (**a**) An example of a round shaped RBC entering a test constriction; (**b**) an example of an elliptical RBC entering a test constriction with the principal axis in the horizontal direction; (**c**) an example of an elliptical RBC entering a test constriction with the principal axis in the vertical direction; and (**d**) the percentages of cell shape and orientation in five subjects.

Different RBC shapes and orientations were found during the data analysis. Figure 9a–c show three types of RBCs with illustrations on the top. The three types are round shaped RBCs, elliptical RBCs with principal axes in the horizontal direction and elliptical RBCs with principal axes in the vertical direction, respectively. The cell shape is determined based on the ratio between the length of first and second principal axes as $\frac{d_{2}}{d_{1}}$. An RBC is defined as round-shaped if its ratio is greater than or equal to 0.9, and the rest are categorized as elliptical RBCs. The entering direction is determined based on cell orientation before contacting the walls of the constrictions. If an elliptical RBC enters the constriction along its first principal axis, the longer side, the RBC is defined as being in a horizontal direction, otherwise, the RBC is said to be in a vertical direction. Figure 9d shows the percentages of each cell type where the round RBCs and elliptical RBCs in the horizontal directio are the most dominant among all.

The round RBCs as shown in Figure 9a are well matched to the mechanical model shown in Figure 2b, and it is expected to show reasonable velocity–deformation correlation. On the other hand, the deformation of elliptical RBCs in the horizontal or vertical direction may need to be considered differently since there is a possible difference in deformation mechanism, that is, the equivalent diameter, *D*, may not be adequate for calculating the deformation, ϵ. For the RBCs like Figure 9b, the amount of deformation might be over-estimated since they were compressed on the short side only. The elliptical RBCs are found from two different conditions after examining experimental videos. One condition is that the RBC is originally shaped as an ellipse while the other condition is that the RBC is originally round, but is pre-deformed by shear stress from the flow before the constrictions. These two cases are obviously different, and are worthy of further investigation. The third type shown in Figure 9c includes the elliptical RBCs entering a constriction with its first principal axis perpendicular to the flow. RBCs in this type were all originally shaped as ellipses, and flowed into the constriction with the vertical orientation. Furthermore, the deformation of RBCs like Figure 9c is more like bending rather than compression in the first two groups as Figure 9a,b. The long side of RBC is folded for entering the constriction, and the mechanism is different from compression along the vertical direction.

**Figure 10:**
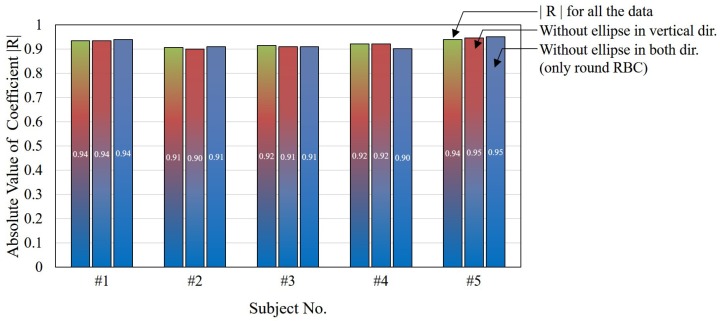
The absolute values of correlation coefficients with and without elliptical RBCs in different orientations.

Figure 10 shows the absolute values of correlation coefficients of all the data, the data without elliptical RBCs in the vertical direction, and the data without all elliptical RBCs from left to right for each subject, respectively. The absolute values of the correlations remain equal to or greater than 0.90. As a result, the shape and orientation of RBCs are not significant for these particular sets of results.

## 6. Conclusions

A microfluidic method for improving the evaluation of RBC deformability is proposed and tested in this paper. Three concluding remarks are: (1) the proposed microfluidic system efficiently provides a wider range of deformation for evaluating representative characteristics of cell deformability with strong correlation; (2) the experimental results on five subjects show that the proposed deformability checker significantly improves the correlation between cell deformation and velocity from an absolute average of |R|=0.54 to |R|=0.92; and (3) different RBC size, shape and orientation are found from experimental results. The differences are discussed through detailed analysis.

multiple

## Supplementary Materials

The following are available online at www.mdpi.com/2072-666X/7/10/176/s1, Video S1: the cell motion through the constrictions of different widths captured by a high-speed camera.
